# The analysis of gene co-expression network and immune infiltration revealed biomarkers between triple-negative and non-triple negative breast cancer

**DOI:** 10.3389/fgene.2024.1505011

**Published:** 2025-01-06

**Authors:** Yao Yi, Yu Zhong, Lianhua Xie, Shuxian Lu, Yifeng Zhang

**Affiliations:** ^1^ Discipline of Chinese and Western Integrative Medicine, Jiangxi University of Chinese Medicine, Nanchang, China; ^2^ Second Affiliated Hospital, Jiangxi University of Chinese Medicine, Nanchang, China; ^3^ Centre for Translational Medicine, Jiangxi University of Chinese Medicine, Nanchang, China

**Keywords:** gene co-expression network, immune infiltration, biomarkers, macrophages, triple negative breast cancer

## Abstract

**Background:**

Triple-negative breast cancer (TNBC) is a heterogeneous disease with a worse prognosis. Despite ongoing efforts, existing therapeutic approaches show limited success in improving early recurrence and survival outcomes for TNBC patients. Therefore, there is an urgent need to discover novel and targeted therapeutic strategies, particularly those focusing on the immune infiltrate in TNBC, to enhance diagnosis and prognosis for affected individuals.

**Methods:**

The gene co-expression network and gene ontology analyses were used to identify the differential modules and their functions based on the GEO dataset of GSE76275. The Weighted Gene Co-Expression Network Analysis (WGCNA) was used to describe the correlation patterns among genes across multiple samples. Subsequently, we identified key genes in TNBC by assessing genes with an absolute correlation coefficient greater than 0.80 within the eigengene of the enriched module that were significantly associated with breast cancer subtypes. The diagnostic potential of these key genes was evaluated using receiver operating characteristic (ROC) curve analysis with three-fold cross-validation. Furthermore, to gain insights into the prognostic implications of these key genes, we performed relapse-free survival (RFS) analysis using the Kaplan-Meier plotter online tool. CIBERSORT analysis was used to characterize the composition of immune cells within complex tissues based on gene expression data, typically derived from bulk RNA sequencing or microarray datasets. Therefore, we explored the immune microenvironment differences between TNBC and non-TNBC by leveraging the CIBERSORT algorithm. This enabled us to estimate the immune cell compositions in the breast cancer tissue of the two subtypes. Lastly, we identified key transcription factors involved in macrophage infiltration and polarization in breast cancer using transcription factor enrichment analysis integrated with orthogonal omics.

**Results:**

The gene co-expression network and gene ontology analyses revealed 19 modules identified using the dataset GSE76275. Of these, modules 5, 11, and 12 showed significant differences between in breast cancer tissue between TNBC and non-TNBC. Notably, module 11 showed significant enrichment in the WNT signaling pathway, while module 12 demonstrated enrichment in lipid/fatty acid metabolism pathways. Subsequently, we identified SHC4/KCNK5 and ABCC11/ABCA12 as key genes in module 11 and module 12, respectively. These key genes proved to be crucial in accurately distinguishing between TNBC and non-TNBC, as evidenced by the promising average AUC value of 0.963 obtained from the logistic regression model based on their combinations. Furthermore, we found compelling evidence indicating the prognostic significance of three key genes, KCNK5, ABCC11, and ABCA12, in TNBC. Finally, we also identified the immune cell compositions in breast cancer tissue between TNBC and non-TNBC. Our findings revealed a notable increase in M0 and M1 macrophages in TNBC compared to non-TNBC, while M2 macrophages exhibited a significant reduction in TNBC. Particularly intriguing discovery emerged with respect to the transcription factor FOXM1, which demonstrated a significant regulatory role in genes positively correlated with the proportions of M0 and M1 macrophages, while displaying a negative correlation with the proportion of M2 macrophages in breast cancer tissue.

**Conclusion:**

Our research provides new insight into the biomarkers and immune infiltration of TNBC, which could be useful for clinical diagnosis of TNBC.

## Introduction

Breast cancer (BC) is the most prevalent malignancy affecting women globally and remains a significant contributor to cancer-related deaths. Over 3 million new cases of breast cancer and 1 million deaths will occur each year worldwide by the year 2040 (Arnold et al., 2022). TNBC constitutes a particularly aggressive and invasive subtype characterized by the absence of estrogen receptor (ER), progesterone receptor (PR), and human epidermal growth factor receptor 2 (HER2) expression (Agarwal et al., 2016). TNBC occurs in about 15%–20% of all breast cancer, which are different from non-TNBC with prognosis and therapeutic targets. Compared with other breast cancer subtypes, TNBC is always related to worse prognosis and lower overall survival rate (Dietze et al., 2015). The lack of hormone receptors and HER2 expression can make it more difficult to cure so that making therapeutic targeting difficult and combination therapy is needed (Wang Y. et al., 2019; Dong et al., 2018). The current main treatment of TNBC is still chemotherapy, but a significant number of patients are drug resistant, leading to poor therapeutic effect, which was not satisfactory (Hu et al., 2023; Yang et al., 2021). Consequently, the identification of novel therapeutic targets holds paramount importance in augmenting the prognosis and treatment efficacy of TNBC (Zhu et al., 2023).

Weighted gene co-expression network analysis (WGCNA) has demonstrated wide-ranging applications in biomarker discovery, including, but not limited to, laryngeal cancer (Liu et al., 2019), lung cancer (Wang S. et al., 2023) and advanced gastric cancer (Wang W-J. et al., 2019). Previous studies have revealed that AMD1, EN1, and VGLL1 are likely to contribute to breast cancer progression and an unfavorable prognosis (Yang et al., 2021). Additionally, IL6ST, HMGA1, FOXM1, and MYBL2 have been identified as potentially playing an important role in TNBC progression using the TNBC gene expression dataset GSE76275 (Jia et al., 2021; Fiscon et al., 2021). Despite this progress, the potential biomarkers and their mechanisms involved in breast cancer subtypes have not been fully elucidated. Furthermore, the involvement of these potential marker genes in tumor-associated macrophages (TAMs) has not been reported. TAMs, one of the main cell types in the tumor immune microenvironment, play a pivotal role in cancer progression (Wang XQ. et al., 2023; Chen et al., 2023). TAMs are innate immune effector cells that are recruited to tumor tissues, contributing to tumor growth and metastasis by promoting angiogenesis and suppressing adaptive immunity (Niu et al., 2016). TAMs represent a heterogeneous and plastic population, within which polarized TAMs can be identified as M1-and M2-like macrophages (Gordon and Taylor, 2005). Recently, some clinical and experimental research has discovered that M1-like macrophages release proinflammatory cytokines and chemokines, such as tumor necrosis factor (TNF-α), interleukin (IL-1β), and CXCL10, exerting antitumor activity (Pe et al., 2022). In contrast, M2-like macrophages produce a high number of anti-inflammatory factors, which may lead to the immune escape of tumor cells and contribute to breast cancer progression (Zhang et al., 2024). The immunosuppressive role of M2 macrophages in TNBC indicates their potential as therapeutic biomarkers (Zhang et al., 2024). However, differences between M1-and M2-like macrophages in TNBC and non-TNBC, especially the biomarkers of M1-and M2-like macrophages in TNBC, are rarely reported. Consequently, a comprehensive characterization of the immune infiltrate in TNBC holds significant promise for identifying patients most likely to benefit from immunotherapy and identifying resistance factors that could serve as potential therapeutic targets.

In this study, we conducted a comprehensive investigation aimed at elucidating the molecular characteristics and immune microenvironment of breast cancer subtypes, with a particular focus on TNBC and non-TNBC. To begin, we employed the gene co-expression network and gene ontology analyses to identify the differential modules and the function of these modules based on the GEO dataset of GSE76275. Next, we identified key genes associated with these subtypes and evaluated their potential as diagnostic biomarkers through ROC curve analysis. Furthermore, we performed relapse-free survival (RFS) analysis on these key genes using the Kaplan-Meier plotter online tool to determine their prognostic value and clinical relevance. Additionally, we estimated the immune cell compositions in breast cancer tissue between TNBC and non-TNBC using the CIBERSORT algorithm. Finally, we identified key transcription factors involved in macrophage infiltration and polarization in breast cancer through transcription factor enrichment analysis by integrating orthogonal omics data.

## Materials and methods

### Data collection and processing

The GSE76275 dataset was retrieved from the GEO database and comprises 198 triple-negative breast cancer tissue samples and 67 non-triple-negative breast cancer tissue samples (Burstein et al., 2015). To ensure complete and high-quality data for subsequent analyses, a filtering procedure was carefully conducted to exclude entries lacking age and BMI information. This rigorous filtering step ensured that only samples with the necessary clinical attributes were retained for further investigation, thereby enhancing the reliability and robustness of our analysis.

### Weighted gene co-expression network analysis (WGCNA)

Weighted gene co-expression network Analysis (WGCNA) for microarray data was constructed and analyzed using the WGCNA package in R (Zhang and Horvath, 2005). In this study, we utilized 20,529 genes to construct the gene co-expression network. Confounding factors, including age and BMI, were adjusted for in the construction of the gene co-expression network. A power threshold of 8 was selected to calculate the weighted adjacency matrix, with the thresholding parameter defined using a scale-free topology with a cutoff R^2^ = 0.8. This cutoff was chosen to be the closest to or slightly above 0.8 to establish a biologically meaningful and stable network for further analysis. We identified gene modules using the ‘hybrid’ method with parameters mergeCutHeight = 0.25 and minModuleSize = 40. Modules were identified as branches in the dendrogram using the Dynamic Tree Cut algorithm (Rezaie et al., 2023). Subsequently, we used topological overlap measures (TOM) to reveal the interconnectedness and functional relationships among genes within the network (Li and Horvath, 2007).

### Evaluation of immune cells between TNBC and Non-TNBC

To identify the different immune cell compositions of breast cancer tissue between TNBC and non-TNBC, we employed the CIBERSORT algorithm, using the leukocyte signature matrix (LM22) as the reference gene expression signatures (Newman et al., 2015). CIBERSORT is an algorithm that utilizes a reference gene expression signature matrix (LM22), representing 22 immune cell types, to deconvolve complex tissue expression data into estimated immune cell fractions (Newman et al., 2015). It employs support vector regression to minimize errors in predicting cell type fractions, even when cell types have highly similar gene expression profiles. We obtained bulk-RNA sequencing data from breast cancer tissue for TNBC and non-TNBC from the GSE76275 dataset for our analysis. Using R software, we fully analyzed and visualized the abundance and proportion of immune cell members among different groups.

### GO and KEGG enrichment analyses

The Gene Ontology (GO) and Kyoto Encyclopedia of Genes and genomes (KEGG) pathway enrichment analysis were performed using “clusterProfile” package in R software (Yu et al., 2012). The Benjamin-Hochberg approach was used to correct multiple tests and select the significant terms and pathways. The adjusted p-value <0.05 was used as a threshold of significance for the enriched terms and pathways for target genes.

### Transcription factor enrichment analysis by orthogonal omics integration

To obtain the key transcription factors of genes that associated with macrophage infiltration and polarization in breast cancer, we performed transcription factor enrichment analysis by orthogonal omics integration following Chip-X Enrichment Analysis Version 3 (ChEA3) (Keenan et al., 2019). TF enrichment analysis (TFEA) prioritizes transcription factors based on the overlap between given lists of differentially expressed genes, and previously annotated TF targets assembled from multiple resources. The multiple resources including ChIP-seq experiments from ENCODE, ReMap, and individual publications; co-expression of TFs with other genes based on processed RNA-seq from GTEx and ARCHS4; co-occurrence of TFs with other genes by examining thousands of gene lists submitted to the tool Enrichr; and gene signatures resulting from single TF perturbations followed by genome-wide gene expression experiments.

### Prognostic analysis of triple negative breast cancer

A Kaplan Meier plotter (https://kmplot.com) database, discovery based on meta-analysis, and validation of online survival biomarker tools were used to study the survival of TNBC in GEO, the European Archives of Genomic Phenomena (EGA), TCGA, and METRIC databases.

### Protein-protein interaction network analysis

The search tool for retrieving interacting genes is a database of known and predicted protein-protein interactions that can be used to predict and track the protein–protein interactions network. This study used the STRING database to construct the PPI network of macrophage infiltration and polarization related genes in breast cancer.

### Statistical analysis

Data statistical analysis and visualization were performed using R software. The correlation among different continuous variables were obtained by Spearman’s correlation coefficient. Multiple comparisons between categorical variables using ANOVA analysis, and t.test were applied for statistical analysis between different groups. The p-value <0.05 was considered as significantly statistical difference in this study.

## Results

### Preprocessing of data and descriptive statistics analysis

**FIGURE 1:**
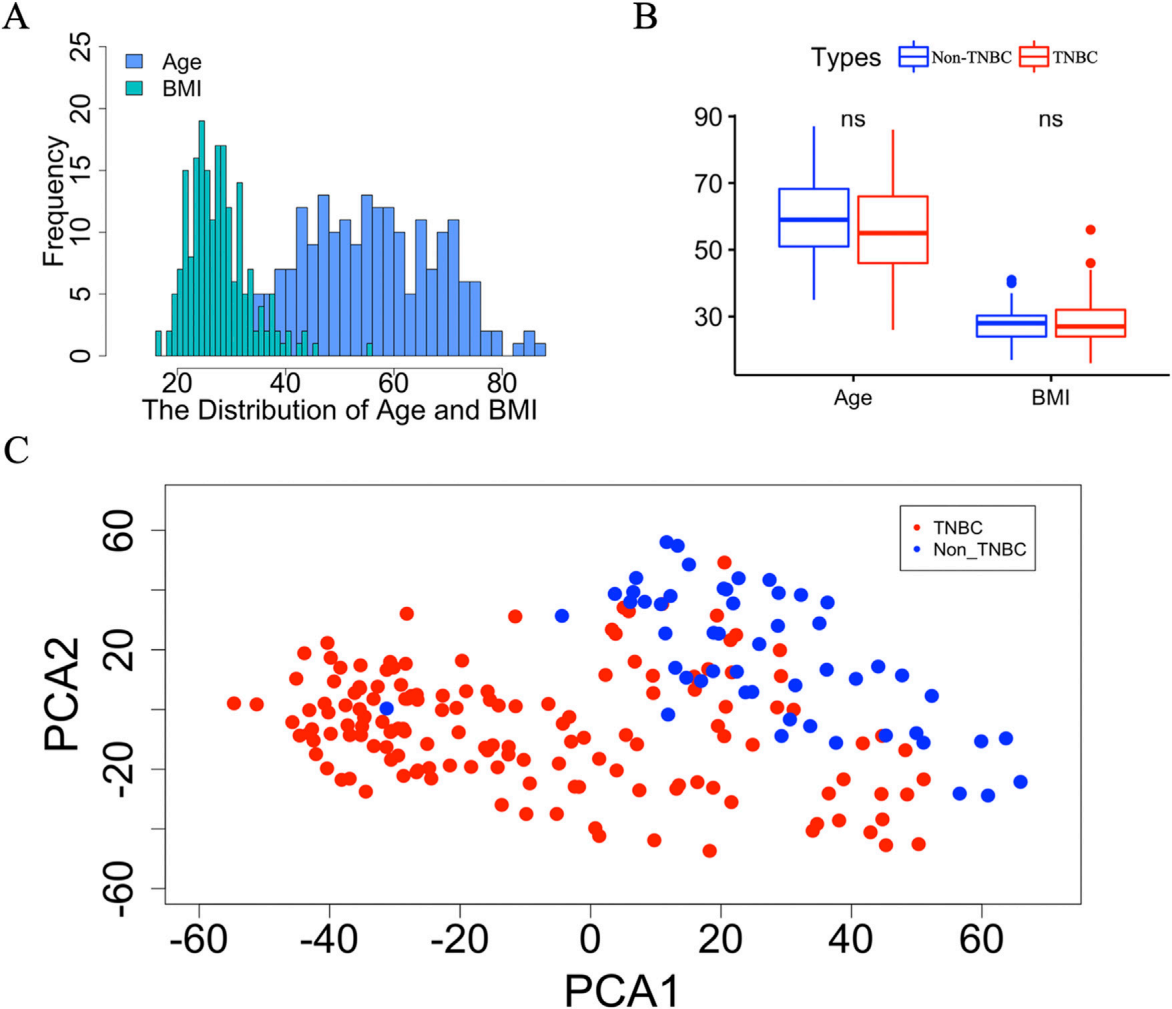
The detail information of breast cancer tissue between TNBC and non-TNBC. **(A)** The distribution of age and BMI in TNBC and non-TNBC. **(B)** Boxplot of age and BMI between TNBC and non-TNBC. The “ns” represents there is no significant difference between Non-TNBC and TNBC group. **(C)** Scatter plot of the first two principal component vectors of the gene expression profiles of samples from TNBC and non-TNBC, which are highlighted using different colors. Red represents TNBC, blue represents non-TNBC.

To obtain more complete clinical information on TNBC and non-TNBC, we removed samples with incomplete age and BMI data. A total of 201 samples, comprising 149 TNBC and 52 non-TNBC samples, were retained for subsequent analyses. All the samples were recorded age (average: 56.5, range: 26-87) and BMI (average: 28.1, range: 16-56) (Figure 1A; Supplementary Table S1). To assess any potential disparities in age and BMI between TNBC and non-TNBC breast cancer tissues, we conducted a comparative analysis, which indicated no significant differences in age or BMI distribution between the two subtypes (Figure 1B). To control for the effect of age and BMI on gene expression, we used a linear mixed model to correct for confounding factors, including age and BMI. After correction, age and BMI had minimal effect on gene expression, thus preserving the integrity of our downstream gene expression analysis (Supplementary Figure S1). Lastly, to examine global gene expression patterns between TNBC and non-TNBC, we conducted principal component analysis (PCA) on the GSE76275 dataset comprising 20,529 genes (Figure 1C). The PCA revealed distinct clustering of TNBC and non-TNBC breast cancer tissue, underscoring substantial differences in gene expression profiles between these two breast cancer subtypes.

### Identification of the TNBC related gene co-expression modules

**FIGURE 2:**
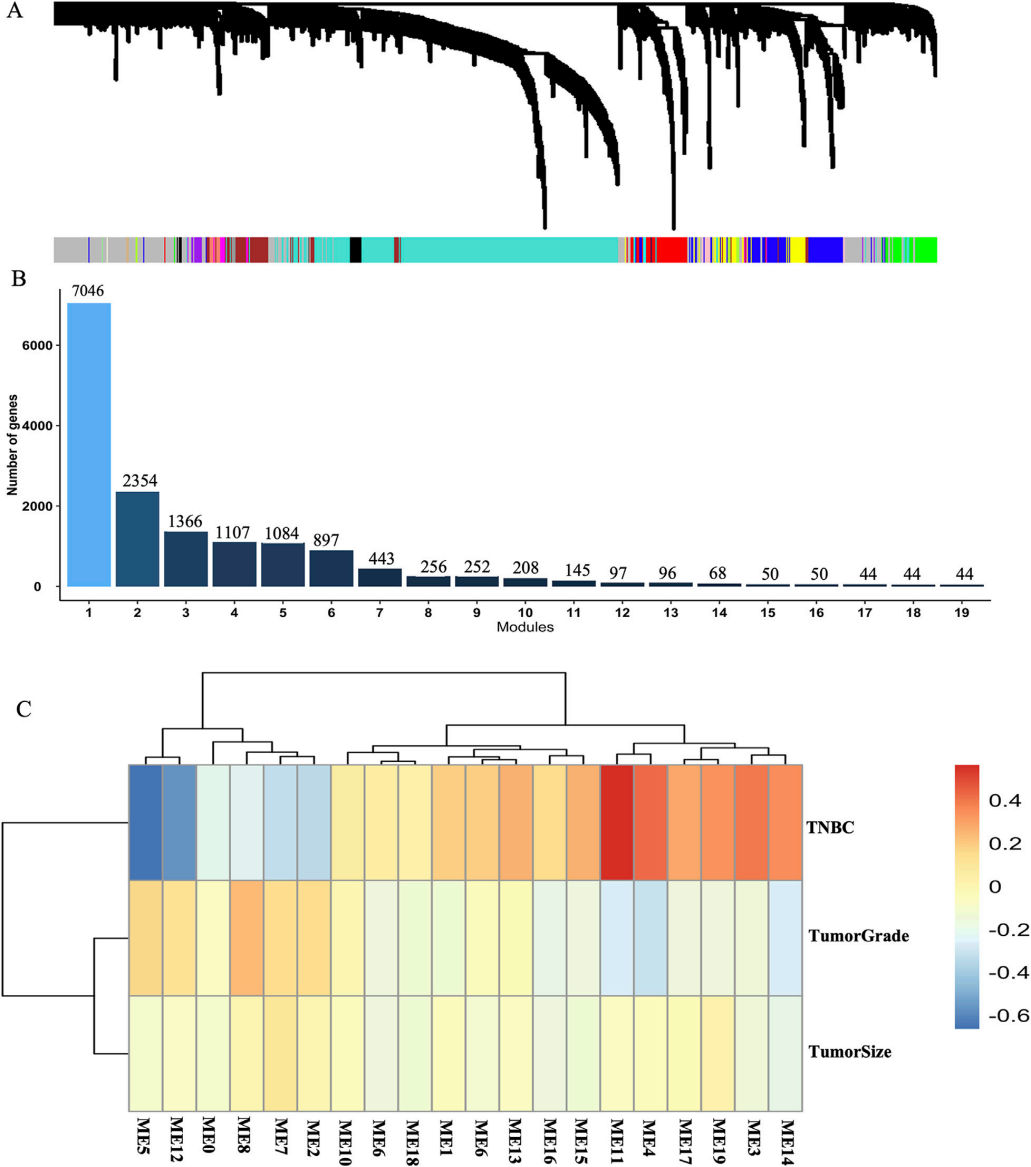
Weighted gene co-expression network constructed in breast cancer tissue between TNBC and non-TNBC. **(A)** Gene co-expression network module in breast cancer tissue between TNBC and non-TNBC. **(B)** The numbers of modules and genes in weighted gene co-expression network modules based on the dataset of GSE76275. **(C)** A heatmap of the correlations between 19 module principal components (PCs) and 3 cancer-related traits including tumor grade, tumor size and the subtypes of breast cancer.

To elucidate the potential functions and underlying mechanisms of key genes in breast cancer tissue between TNBC and non-TNBC, we constructed a weighted gene co-expression network using the GSE76275 dataset. For creating a robust co-expression network, we applied a R^2^ cutoff of 0.80 and set the soft-threshold power (β) to 8. Consequently, we constructed adjacency matrices to capture comprehensive co-expression information across the network. This analysis successfully derived 19 gene modules, each containing more than 40 genes (Figures 2A, B). Subsequently, we built additional co-expression networks associated with tumor grade, tumor size, and breast cancer subtypes by employing the Pearson correlation coefficient within these 19 gene modules through WGCNA analysis. Our results revealed that modules 5, 11, and 12 displayed significant correlations with the breast cancer subtypes, as evidenced by correlation coefficients greater than 0.5 and adjusted p-values less than 0.05 (Supplementary Table S2). Notably, module 11 also exhibited a significant negative correlation with tumor grade. However, no significant correlations were observed between the gene modules and tumor size (Figure 2C). These findings indicate the potential functional relevance of the identified gene modules in breast cancer pathogenesis and further underscore the importance of these genes in distinguishing between TNBC and non-TNBC subtypes.

### Function annotation and key genes of TNBC related modules

**FIGURE 3:**
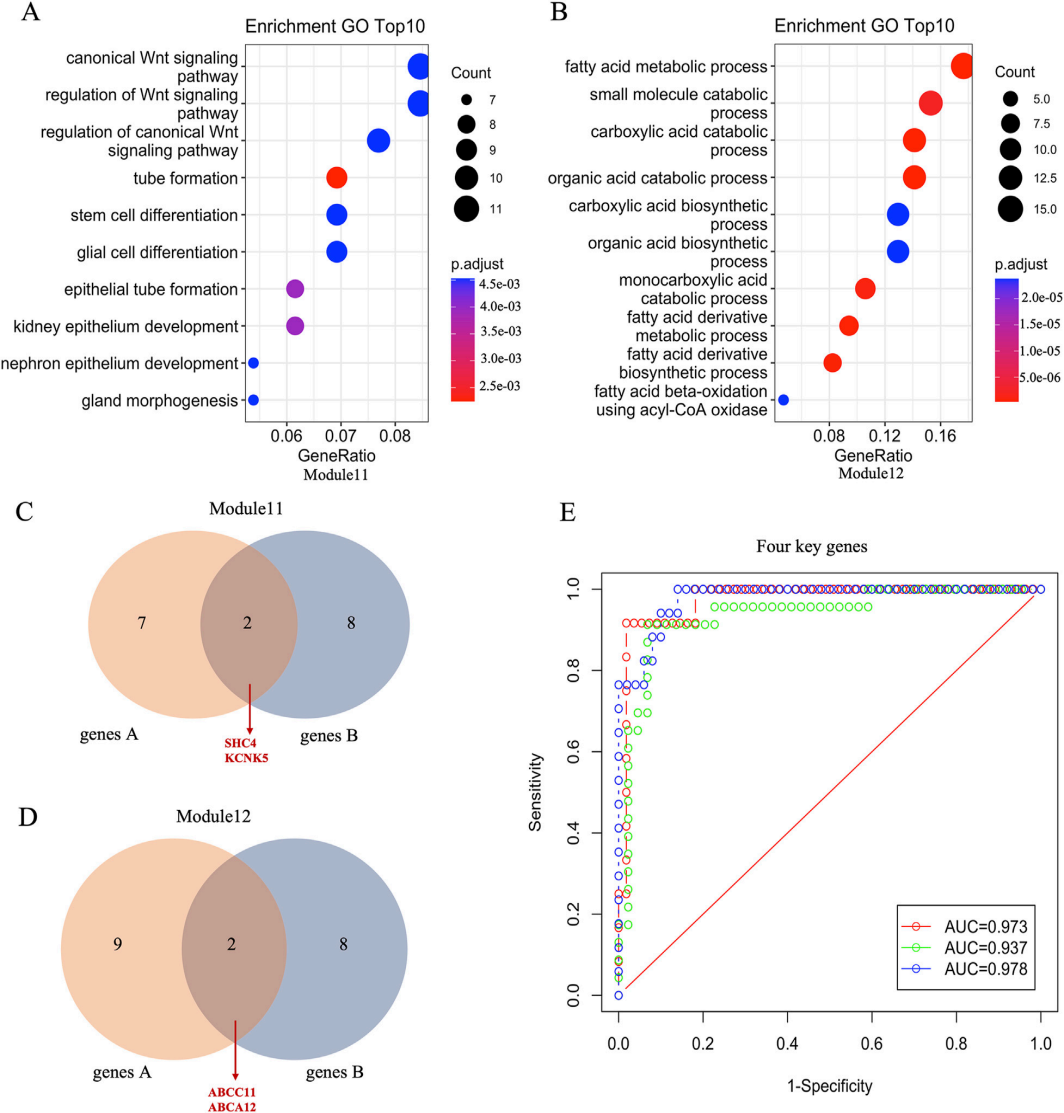
Function annotation and key genes of TNBC related modules. **(A, B)** The top 10 enrichment pathways of genes in module11 and module12. **(C, D)** Venn diagrams showing common genes between genes A and genes B in module 11 and module 12, respectively. Genes A represents with exhibit an absolute correlation coefficient >0.80 within the eigengene of the enriched module and significantly correlated with the subtypes of breast cancer, and Genes B represents top 10 genes significantly associated with the subtypes of breast cancer. **(E)** The ROC curve of four essential gene combinations is based on the logistic regression model.

Next, we performed bioinformatics analysis of breast cancer-related modules using clusterProfiler package in R software. The results highlighted two distinct modules, module 11 and module 12, each associated with specific biological processes (Figure 3A; Supplementary Table S3). Genes within module 11 were found to be involved in the regulation of canonical Wnt signaling, tube formation, and epithelium development, while those in module 12 were primarily associated with fatty acid metabolic processes, small molecule catabolic processes, and organic acid catabolic processes. (Figure 3B; Supplementary Table S3). These results indicated that these pathways are essential to involve in the progression of TNBC. Moreover, we identified 9 genes within the enriched modules by selecting those with module membership (MM) absolute correlation coefficients greater than 0.80, and significant correlations with breast cancer subtypes in module 11. The overlapping genes between these 9 genes and the top 10 genes with significant correlation with breast cancer subtypes were considered as key genes in module 11. The identification process for the 2 key genes in module 12 is consistent with that used for identifying key genes in module 11. Specifically, SHC4 and KCNK5 were identified as crucial key genes in module 11, while ABCC11 and ABCA12 were identified as crucial key genes in module 12 (Figures 3C, D). Notably, SHC4 and KCNK5 exhibited significantly higher gene expression levels in TNBC compared to non-TNBC (Supplementary Figure S2), while ABCC11 and ABCA12 displayed lower expression levels in TNBC (Supplementary Figure S3). Finally, we evaluated the diagnostic potential of these key genes using receiver operating characteristic (ROC) curve analysis with three-fold cross-validation. The results showed that the AUC of the three verification sets in the logistic model constructed by the 3-fold cross-validation method was 0.973, 0.937 and 0.978 with an average AUC of 0.963 (Figure 3E). Taken together, these results suggest that these four key genes play key roles in the progression of TNBC which may contribute potential targets for the diagnosis, treatment, and prognosis assessment of TNBC.

### The association between key targets and prognosis of TNBC

**FIGURE 4:**
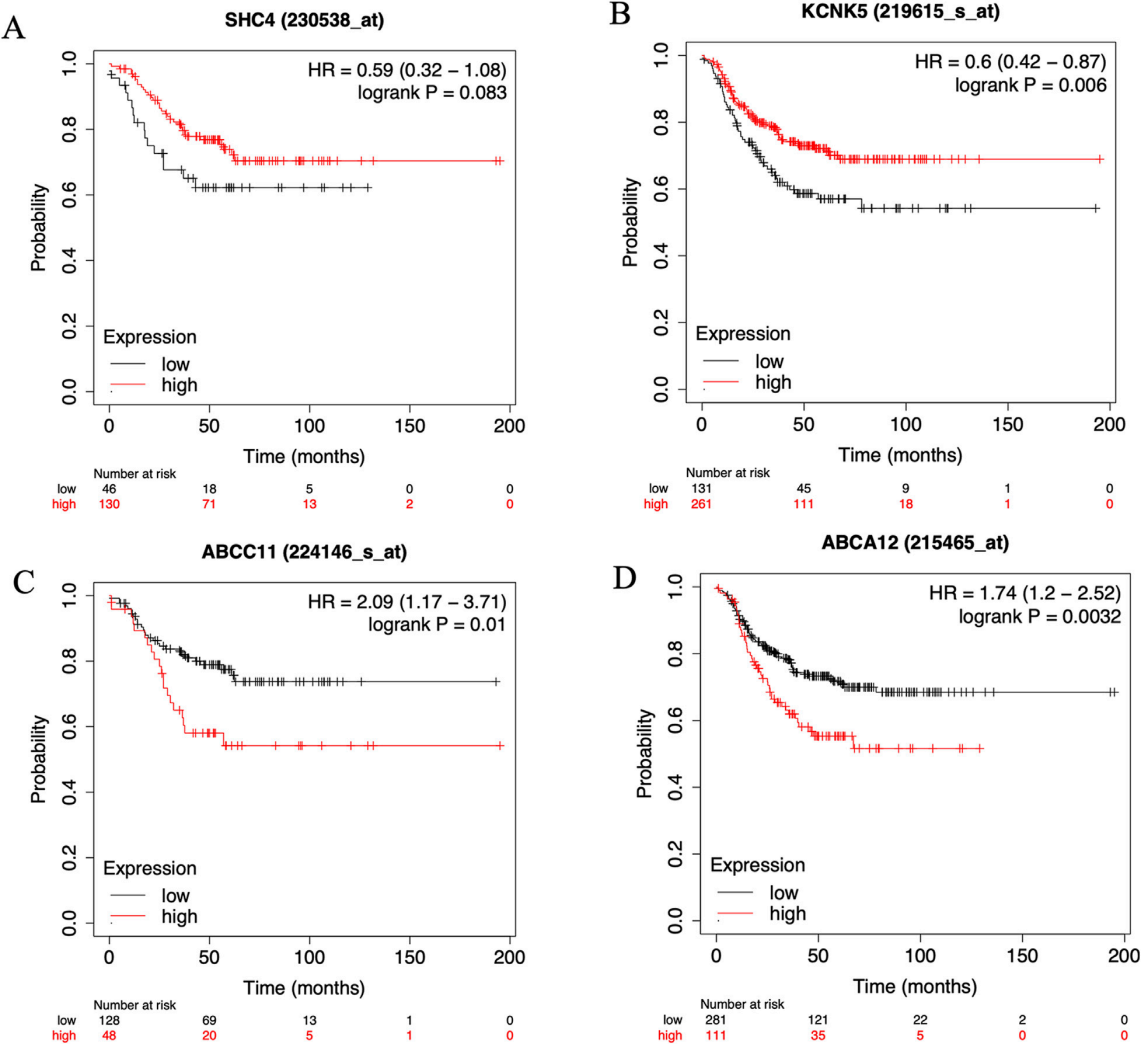
The KM plotter online cancer survival analysis tool (http://kmplot.com/analysis/) to evaluate the Relapse-free survival (RFS) in six key genes. **(A)** *SHC4*, **(B)** *KCNK5,* **(C)** *ABCC11* and **(D)** *ABCA12* in samples with TNBC on Kaplan Meier plotter analysis.

To elucidate the prognostic implications of the four identified genes in TNBC, we performed a comprehensive prognostic analysis using the Kaplan-Meier plotter online tool. The results indicated that three out of the four genes significantly impact TNBC prognosis, as shown in Figure 4. Specifically, KCNK5, ABCC11, and ABCA12 emerged as key prognostic markers with substantial implications for clinical management and treatment decisions in TNBC (Figure 4). These findings underscore the potential clinical relevance of these three target genes in predicting patient outcomes and highlight their utility as prognostic indicators in TNBC.

### Identification of the immune cell compositions between TNBC and Non-TNBC

**FIGURE 5:**
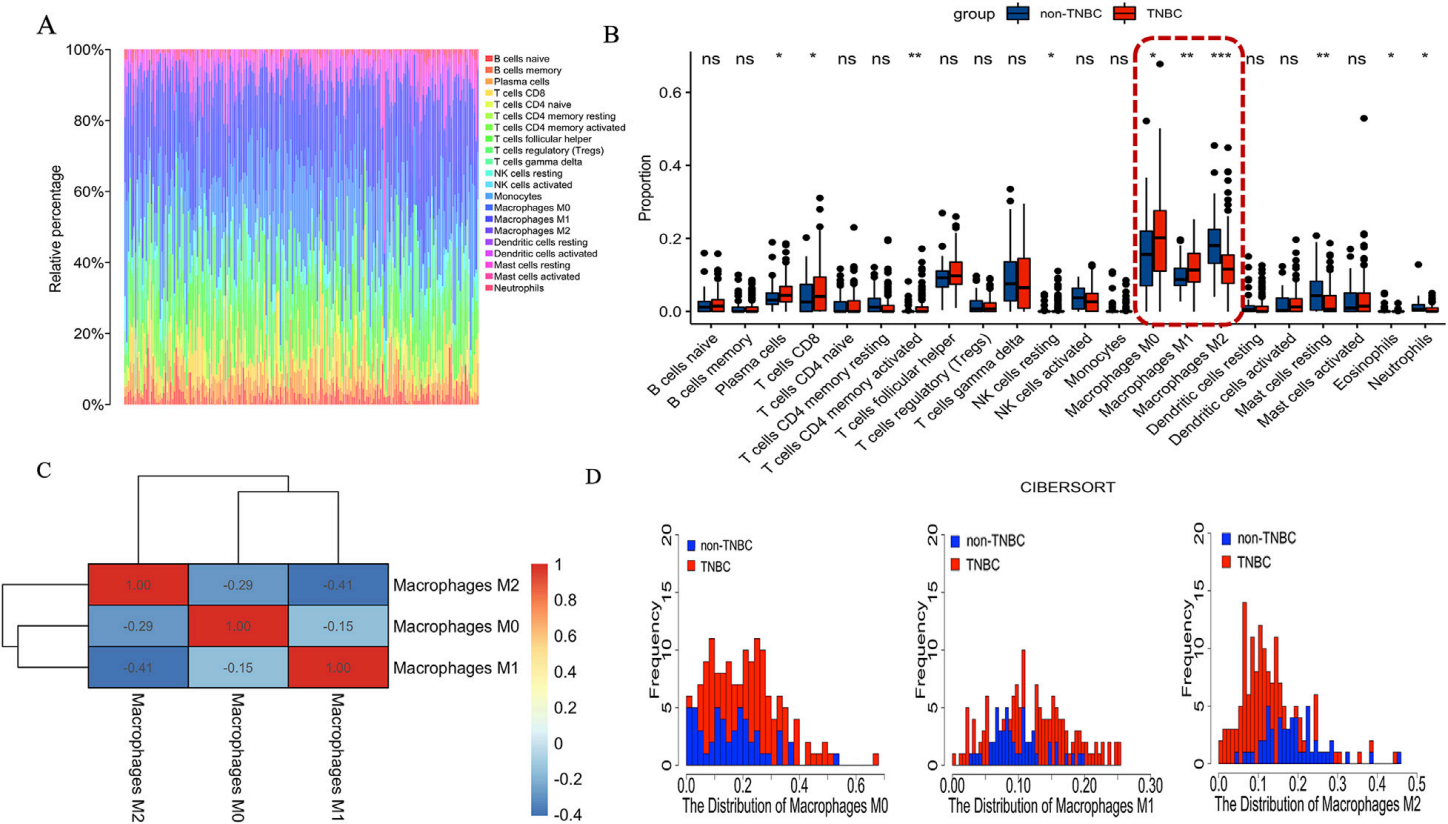
The immune cell compositions in breast cancer tissue between TNBC and non-TNBC. **(A)** The fraction of 22 subsets of immune cells in breast cancer tissue between TNBC and non-TNBC. **(B)** The violin graph shows the difference of immune infiltration between TNBC and non-TNBCs. Red represents TNBC, Blue represents non-TNBC. **(C)** Pearson correlation coefficient between the different macrophages in breast cancer tissue. Red represents positive correlation. Blue represents negative correlation. **(D)** The proportion of distribution of different macrophages in breast cancer tissue.

The CIBERSORT deconvolution algorithm was exploited to access the immune cell compositions in breast cancer tissue between TNBC and non-TNBC. We summarized the results obtained from the remaining 201 breast cancer tissue between TNBC and non-TNBC in Figure 5A. Compared to breast cancer tissue of non-TNBC, the breast cancer tissue in TNBC exhibited higher infiltration of M0 and M1 macrophages, lower infiltration of M2 macrophages. In addition to macrophages, there were seven immune cell types also exhibiting significantly differential proportions in breast cancer tissue between TNBC and non-TNBC including plasma cells, T cells CD8, T cells CD4 memory activated, NK cells resting, mast cells resting, eosinophils and neutrophils (Figure 5B). Nonetheless, none of these immune cell types exhibited proportions greater than those of macrophages, suggesting that the infiltration and polarization of tumor-related macrophages are crucial distinguishing features in breast cancer tissue between TNBC and non-TNBC. Additionally, we calculated the Pearson correlation coefficient between different macrophages and found predominantly negative and significant correlations in breast cancer tissue between TNBC and non-TNBC (Figure 5C). Finally, we depicted the distribution of different macrophage types in TNBC and non-TNBC breast cancer tissues (Figure 5D). On average, TNBC samples showed higher proportions of M0 macrophages (20.5%) and M1 macrophages (12.1%) compared to non-TNBC, where M0 and M1 macrophages averaged 15.9% and 9.79%, respectively. Conversely, the average proportion of M2 macrophages in TNBC (13.0%) was notably lower than in non-TNBC (18.6%). Together, these findings highlight macrophage infiltration and polarization as crucial differentiating features between TNBC and non-TNBC breast cancer tissue.

### Identification of transcription factors associated with immune infiltration

**FIGURE 6:**
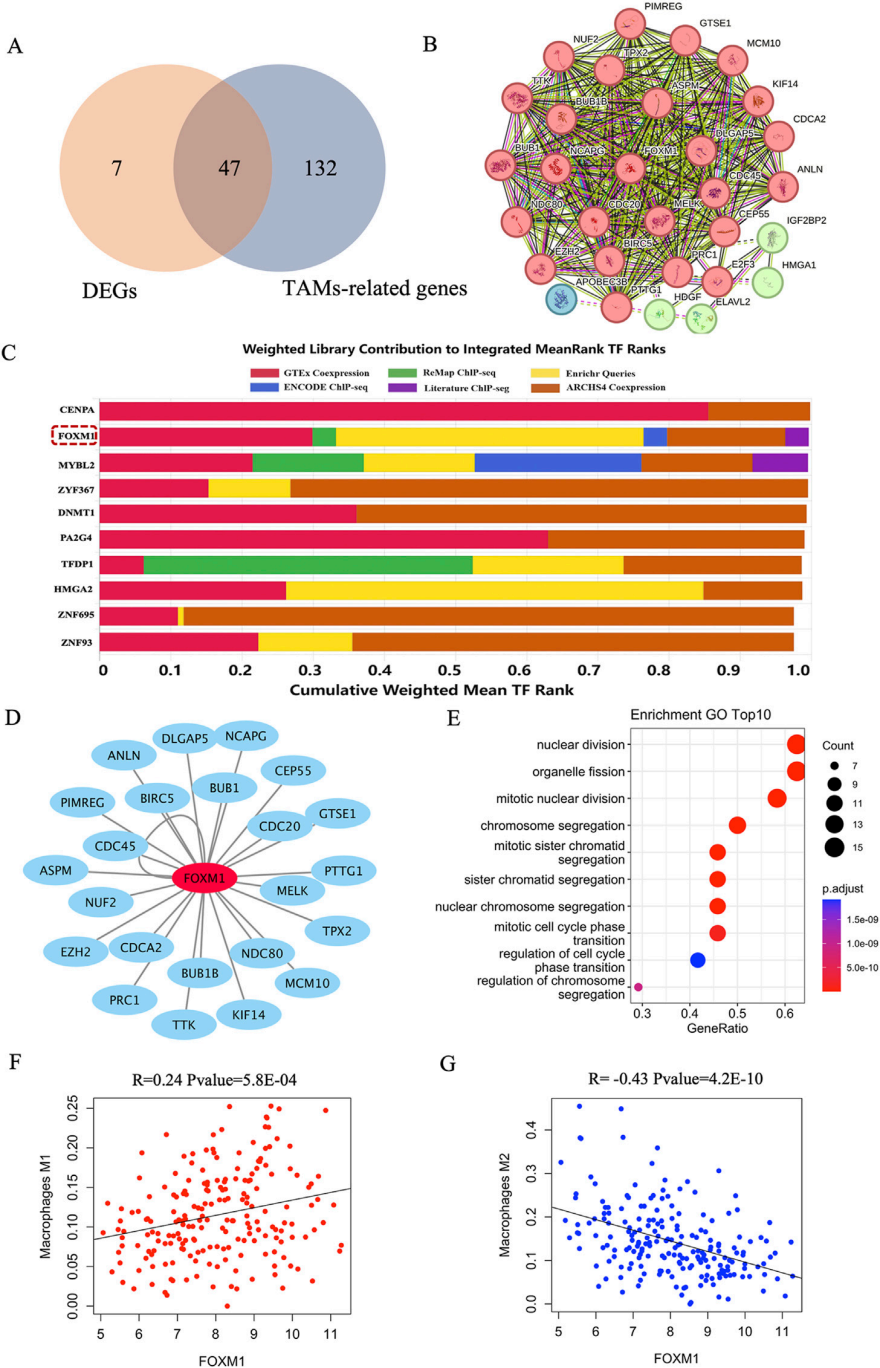
The key transcription of macrophage infiltration and polarization in breast cancer tissue. **(A)** The intersection of DEGs and TAMs-related genes in breast cancer tissue. DEGs: the differential expression genes in breast cancer tissue between TNBC and non-TNBC. **(B)** PPI analysis of the intersection genes of DEGs and TAMs-related genes. **(C)** The top 10 transcription factors of 24 genes with high connectivity. **(D)** Visualization of FOXM1 and their corresponding target gene networks. **(E)** The top 10 enrichment pathways of target genes of FOXM1. **(F)** The correlation between FOXM1 and the proportion of M1 macrophages. **(G)** The correlation between FOXM1 and the proportion of M2 macrophages.

To identify transcription factors that affect macrophage infiltration and polarization in breast cancer tissue, we first identified 179 genes displaying a positive correlation with the proportions of M0 and M1 macrophages, while concurrently exhibiting a negative correlation with the proportion of M2 macrophages. Notably, 47 of these genes demonstrated significant differential expression between TNBC and non-TNBC samples (Figure 6A). Subsequently, a Protein-Protein Interaction (PPI) network analysis was conducted to explore the interaction patterns among the 47 differentially expressed genes, resulting in the identification of 24 genes exhibiting substantial connectivity degrees based on the STRING database (Figure 6B). These results indicated that these 24 genes were considered potentially crucial elements in the network associated with tumor-associated macrophages (TAMs) in breast cancer tissues, specifically distinguishing between TNBC and non-TNBC. Further investigations focused on transcription factor enrichment analysis, which unveiled the top 10 transcription factors governing the expression of the 24 identified genes. Among these, FOXM1 stood out due to its significant differential expression in breast cancer tissues between TNBC and non-TNBC, being notably upregulated in TNBC samples (Figure 6C; Supplementary Figure S4). Finally, we conducted functional enrichment analysis on the genes regulated by FOXM1 and found that they were significantly enriched in the nuclear division, organelle fission, and chromosome segregation pathway (Figure 6E). Notably, a substantial positive correlation emerged between the proportion of FOXM1 and M1 macrophages, while conversely, a significant negative correlation was observed with the proportion of M2 macrophages (Figures 6F, G). These results emphasized the crucial role of FOXM1 in the progression of TNBC, modulating genes significantly associated with macrophage infiltration and polarization.

## Discussion

The subtypes of breast cancer not only have different clinical manifestations, but also have different prognostic significance. Among these subtypes, triple-negative breast cancer (TNBC) has garnered significant attention in both clinical and experimental research. Due to its high-risk biological features and limited specific treatment options. Consequently, there is a pressing need to gain a deeper understanding of TNBC biology to identify potential therapeutic targets. In this study, we aimed to recognize meaningful prognostic biomarkers for TNBC by analyzing the GSE76267 dataset using bioinformatic methods. Firstly, we constructed weighted gene co-expression networks based on gene expression profiles of breast cancer tissue, considering 149 TNBC and 52 non-TNBC cases. By identifying key genes and modules within these networks, we sought to pinpoint specific genes associated with TNBC prognosis. The prognostic significance of these key targets was validated using the Kaplan-Meier tool, providing valuable insights into their potential clinical relevance. Furthermore, we also investigated the immune cell composition of breast cancer tissue between TNBC and non-TNBC. Additionally, we conducted an in-depth analysis to identify the transcription factors responsible for regulating genes associated with macrophage infiltration and polarization in breast cancer tissue.

Utilizing WGCNA, we diligently investigated the associations between gene modules and subtypes of TNBC. Our analysis revealed a substantial correlation between modules 5, 11, and 12 and the specific TNBC subtypes, as evidenced by correlation coefficients exceeding 0.5 and adjusted p-values below 0.05. These compelling findings indicate that the genes encompassed within these modules play pivotal roles in the intricate progression of TNBC. Of particular interest, module 11 emerged as an enriched hub of biological processes, encompassing the regulation of canonical Wnt signaling, tube formation, and epithelium development. Notably, each of these processes has been extensively implicated in the pathogenesis of TNBC. For instance, Wnt signaling regulates a variety of cellular processes, including cell fate, differentiation, proliferation, and stem cell pluripotency. Perturbations in Wnt signaling have been implicated in the progression of TNBC, signifying its functional relevance in this aggressive breast cancer subtype (Pohl et al., 2017). In previous studies, it has been reported that tube-formation assay disclosed the function of PCAT6 on angiogenesis (Dong et al., 2020). These results indicated that tube formation mediated angiogenesis is involved in the progression of TNBC. Concurrently, module 12 emerged as a vital repository of genes primarily involved in fatty acid metabolic processes, small molecule catabolic processes, and organic acid catabolic processes. The results demonstrated that fatty acid metabolic process might be potential differential pathways in breast cancer tissue between non-TNBC and TNBC. Fatty acid synthesis and fatty acid oxidation are generally viewed as counterparts in metabolic reprogramming of tumor cells (Ma et al., 2018). In previous reported that inhibition of fatty acid oxidation as a therapy for MYC-overexpressing triple-negative breast cancer. In summary, our rigorous WGCNA analysis unraveled distinct gene modules significantly correlated with specific TNBC subtypes, thereby illuminating key biological processes pertinent to the intricate landscape of TNBC progression. The identification of these critical pathways and their potential therapeutic implications may inform the development of targeted treatments and precision medicine approaches, thus holding promise for improving clinical outcomes in TNBC patients.

Our analysis pinpointed SHC4 and KCNK5 as essential genes within module 11, exhibiting significant correlations with the subtypes of breast cancer. These findings strongly suggested that SHC4 and KCNK5 play crucial roles in the progression of TNBC. The Src homology and collagen (SHC) family is one of the most studied adaptor protein families consisting of four members, SHC1, SHC2, SHC3, and SHC4 (Ahmed and Prigent, 2017). SCH4 could promote tumor proliferation and metastasis by activating STAT3 signaling in hepatocellular carcinoma (Zhang et al., 2022). While SHC4 has been implicated in promoting tumor proliferation and metastasis by activating STAT3 signaling in hepatocellular carcinoma, its specific involvement in TNBC remains relatively unexplored, prompting our investigation into its potential role in regulating tumor proliferation and metastasis in TNBC progression. Furthermore, KCNK channels (also known as K2P, for two-pore-domain potassium channels) are potassium-selective channels that tend to be constitutively open (Goldstein et al., 1996). KCNKs may regulate breast cancer progression via modulating immune response which can serve as ideal prognostic biomarkers for breast cancer (Zou et al., 2022). Our results further supported that KCNKs as potential biomarker in triple negative breast cancer. In addition to SHC4 and KCNK5, we also identified ABCC11 and ABCA12 genes as key players associated with the progression of TNBC. ATP-binding cassette (ABC) transporters are membrane proteins that efflux various compounds from cells, including chemotherapeutic agents, and are known to affect multidrug resistance (Locher, 2016). ABC family may be a useful tool in determining personalized TNBC treatment (Makuch-Kocka et al., 2023; Park et al., 2006). In our study further supported ABC family including ABCC11 and ABCA12 genes plays an important role in the progression of TNBC.

To evaluate the discriminative power of the four-gene combination (SHC4, KCNK5, ABCC11, and ABCA12), we utilized the receiver operating characteristic (ROC) curve in a logistic model constructed through 3-fold cross-validation. The resulting average area under the curve (AUC) of 0.963 underscores the potential of these genes as effective discriminators between TNBC and non-TNBC. Moreover, through prognostic analysis using these key markers, we found that KCNK5, ABCC11, and ABCA12 significantly impact the prognosis of TNBC. Importantly, previous studies have already reported KCNK5 and ABCC11 as prognostic signatures in breast cancer. Specifically, high expression levels of ABCC11 were associated with worse disease-free survival in patients with HER2+ and triple-negative tumor subtypes (Yamada et al., 2013). Additionally, KCNK genes have been identified as prognostic signatures for breast cancer, including TNBC (Zou et al., 2022). ABCA12 is a highly expressed gene in cancer tissues and cells and has been identified as a key gene related to the prognosis of TNBC for the first time in our study. These results demonstrated that these four genes might be potential biomarkers for clinical diagnosis of TNBC. Our findings highlight the potential significance of SHC4, KCNK5, ABCC11, and ABCA12 as potential biomarkers for the clinical diagnosis and prognosis assessment of TNBC. These discoveries contribute to a deeper understanding of TNBC biology and hold promise for the development of targeted therapeutic approaches and precision medicine strategies aimed at improving outcomes in TNBC patients.

Growing evidence underscores the role of tumor-infiltrating lymphocytes in this subtype of breast cancer (García-Teijido et al., 2016). Among these immune cells, tumor-associated macrophages (TAMs), derived from blood monocytes, hold significant prominence as they constitute a substantial portion of tumor-infiltrating immune cells and are influenced by factors secreted by both tumor cells and the tumor stroma (Niu et al., 2016). The presence of TAMs has been linked to unfavorable prognosis and aggressive tumor characteristics (Zhang et al., 2016; Zhao et al., 2017). As the tumor microenvironment emerges as a critical target for cancer immunotherapies, comprehending the immune cell composition in TNBC and its implications is of paramount importance. In this study, We observed that TAMs were the predominant immune cell population in both subtypes, a finding consistent with previous research (Huang et al., 2022). M1 macrophages are recognized for their tumor-killing functions, mediated through cancer cell recognition, phagocytosis, and proinflammatory cytokine production (Choi et al., 2018; Yang et al., 2020). Conversely, M2 macrophages have been associated with promoting tumor cell invasion, metastasis, angiogenesis, and facilitating immune system evasion (Yu and Di, 2017; Linde et al., 2018). However, intriguingly, we noted a distinct pattern of macrophage infiltration and polarization between TNBC and non-TNBC. Specifically, TNBC exhibited significantly higher levels of macrophage infiltration and M1 macrophage polarization, whereas M2 macrophage polarization was lower in TNBC compared to non-TNBC. Our findings align with previous studies indicating that M1 macrophage marker genes are significantly increased in TNBC compared to non-TNBC (Pe et al., 2022). This increase may be attributed to the typically high inflammatory state of TNBC, which promotes M1 macrophage polarization via pro-inflammatory cytokines such as TNF-α, IL-6, and IFN-γ. Additionally, TNBC often presents with elevated levels of tumor antigens, which can stimulate the host immune system and recruit M1 macrophages (Li Y. et al., 2022). This immune response can result in a more pro-inflammatory tumor microenvironment, enhancing M1 macrophage infiltration. M2 tumor-associated macrophages are enriched in the TNBC microenvironment, secreting anti-inflammatory factors that inhibit T cell activity, promote immune escape, and support tumor growth (Chang et al., 2024). TAMs are predominant immune infiltrating cells in both TNBC and non-TNBC. Thus, targeting TAMs, particularly by maintaining the balance between M1 and M2 macrophages, is crucial for inhibiting tumor progression and immune evasion. In TNBC, reducing the number of M2 macrophages, hindering the immune escape of tumor cells, and inhibiting tumor progression are potential therapeutic strategies. In this study, we observed that M2 macrophages were significantly more abundant in non-TNBC than in TNBC. This suggests that the higher presence of M2 macrophages in non-TNBC could lead to greater immune evasion compared to TNBC. In non-TNBC, hormone receptor signaling pathways, such as those involving ER or PR, may facilitate M2 macrophage polarization. In contrast, TNBC, which lacks these signals, exhibits a relatively lower proportion of M2 macrophages. Therefore, the immune suppressive environment in non-TNBC, characterized by higher M2 macrophage levels, presents an opportunity for treatments aimed at depleting M2 macrophages or blocking tumor immune escape as a potential targeting strategy. These findings highlight the importance of understanding the macrophage polarization between M1 and M2 macrophages in TNBC and non-TNBC, as it holds significant potential for advancing targeted immunotherapies and personalized treatment approaches.

Following transcription factor enrichment analysis, we identified FOXM1 as a key transcription factor significantly associated with immune cell infiltration in breast cancer. FOXM1 is the sole member of the FOXM subfamily, which is a critical transcription factor for both the G1-S and the G2-M cell cycle transition (Kalathil et al., 2021). Notably, FOXM1 has been implicated in various aspects of cancer initiation and progression, playing crucial roles in tumor angiogenesis, proliferation, migration, invasion, epithelial–mesenchymal transition, metastasis, prevention of premature cellular senescence, and chemotherapeutic drug resistance (Halasi and Gartel, 2013). The oncogenic role of FOXM1 in inducing cell proliferation, motility, invasion, and tumor growth in TNBC tumor models has been established (Hamurcu et al., 2019). The mechanisms by which FOXM1 promotes tumor growth in TNBC mainly include its regulation of the expression and activity of focal adhesion kinase (FAK) in TNBC cells (Hamurcu et al., 2017), and its direct binding to the promoter of the KIF23 gene to promote its transcription and accelerate TNBC progression via the Wnt/β-catenin pathway (Li Z. et al., 2022). Because the upregulation of FOXM1 to high levels is particularly common in TNBC, there is potential for reducing TNBC aggressiveness and metastasis by inhibiting FOXM1 activity. Compounds that inhibit FOXM1 have been shown to suppress TNBC progression and tumor metastasis (Dey et al., 2020). New inhibitors of FOXM1 have been highlighted as an attractive target for controlling drug-resistant and difficult-to-treat breast cancers (Dey et al., 2020). In previous studies, the macrophage-specific deletion of FOXM1 was found to reduce the expression of iNOS, IL-1β, and IL-6 (Balli et al., 2012). Additionally, FOXM1 inhibition in diabetic mouse models was shown to reduce neutrophil and macrophage recruitment to diabetic wounds *in vivo* (Sawaya et al., 2020). These findings indicated that FOXM1 activation could increase the expression of pro-inflammatory cytokines, driving macrophages toward an M1-like polarization. Our study provides additional support for the involvement of FOXM1 in macrophage infiltration and polarization in the context of breast cancer, particularly between TNBC and non-TNBC. By reducing FOXM1 expression, there is a potential decrease in the proportion of M1 macrophages and an increase in the proportion of M2 macrophages. These results suggests that in the future research on FOXM1 inhibitors, we also need to consider the ratio of M1 and M2 macrophages *in vivo* to maintain the balance of TAMs in the tumor microenvironment, which may help develop an attractive drug for controlling drug-resistant and difficult-to-suppress breast cancers.

In summary, this study offers a comprehensive view of immune infiltration differences between non-TNBC and TNBC. However, our study has several limitations. Firstly, the data used is sourced from the GSE76275, which contains a limited number of datasets, potentially leading to bias. Secondly, there may be unaccounted confounding factors, such as age, sex, or treatment history, in the prognostic analysis. Thirdly, the potential biomarkers identified should be validated *in vitro*. Further validation of the biomarkers’ mechanisms may facilitate their application in predictive diagnostics, patient stratification, targeted prevention, and the personalization of medical services for breast cancer.

## Conclusions

Our results indicated that gene co-expression modules 5, 11 and 12 are most significantly associated with the subtypes of breast cancer. We found that modules 5, 11, and 12 exhibited strong associations with the subtypes of breast cancer, with modules 11 and 12 being notably enriched in the WNT signaling pathway and lipid/fatty acid metabolism pathway, respectively. Within these modules, SHC4, KCNK5, ABCC11, and ABCA12 emerged as key genes with significant relevance to the progression of TNBC. These key genes proved to be crucial in accurately distinguishing between TNBC and non-TNBC, as evidenced by the promising average AUC value of 0.963. Of these key genes, KCNK5, ABCC11 and ABCA12 genes have a significant impact the prognosis of TNBC. Moreover, we observed distinctive immune cell compositions between TNBC and non-TNBC, characterized by higher proportions of M0 and M1 macrophages in TNBC and lower proportions of M2 macrophages compared to non-TNBC. A notable discovery from our study was the identification of FOXM1 as a crucial transcription factor significantly associated with macrophage infiltration and polarization in TNBC progression. In the future, we could develop new FOXM1 inhibitors to inhibit the progression of TNBC from the aspects of FOXM1 inhibition and M1 and M2 macrophage balance. We also plan to collect clinical triple-negative breast cancer data for subsequent validation of these specific markers. These findings provide valuable insights into the molecular and immunological aspects of TNBC, potentially serving as a basis for the development of new therapeutic strategies.

## Data Availability

The datasets presented in this study can be found in online repositories. The names of the repository/repositories and accession number(s) can be found in the article/Supplementary Material.

## Abbreviations

TNBC, Triple negative breast cancer; Non-TNBC, Non-triple negative breast cancer; ChEA3, Chip-X Enrichment Analysis Version 3; TAMs, Tumor-associated macrophage; RFS, Relapse-free survival; LM22, Leukocyte gene signature matrix; ER, Estrogen receptor; PR, Progesterone receptor; HER2, Human epidermal growth factor receptor.
