# Supplementary figures and images for: The analysis of gene co-expression network and immune infiltration revealed biomarkers between triple-negative and non-triple negative breast cancer
Source: Front Genet. 2025 Jan 6;15:1505011. doi: 10.3389/fgene.2024.1505011 (PMC11743633; doi:10.3389/fgene.2024.1505011)

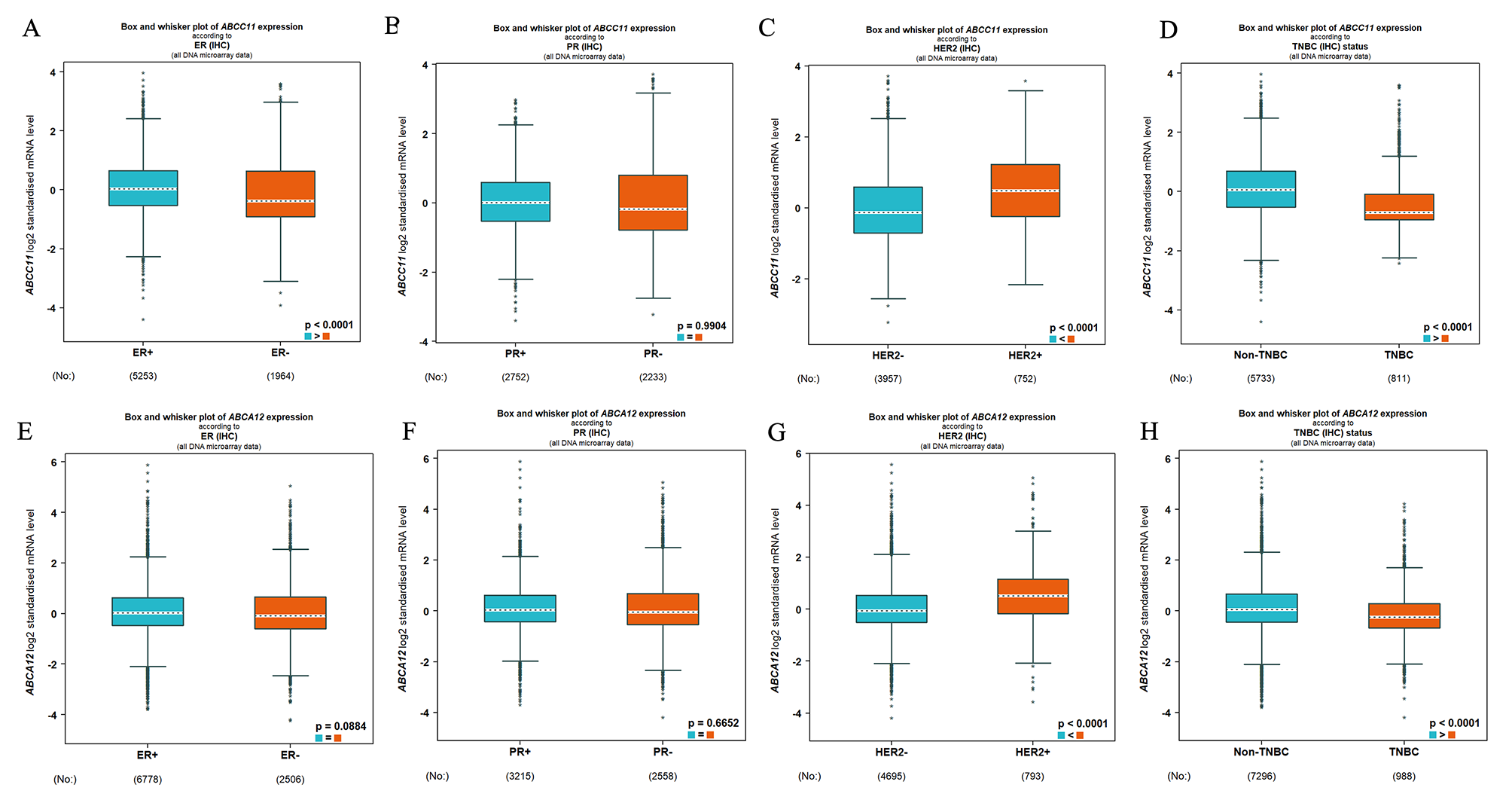

Supplement: Supplementary file 3 [file Image3.TIF]

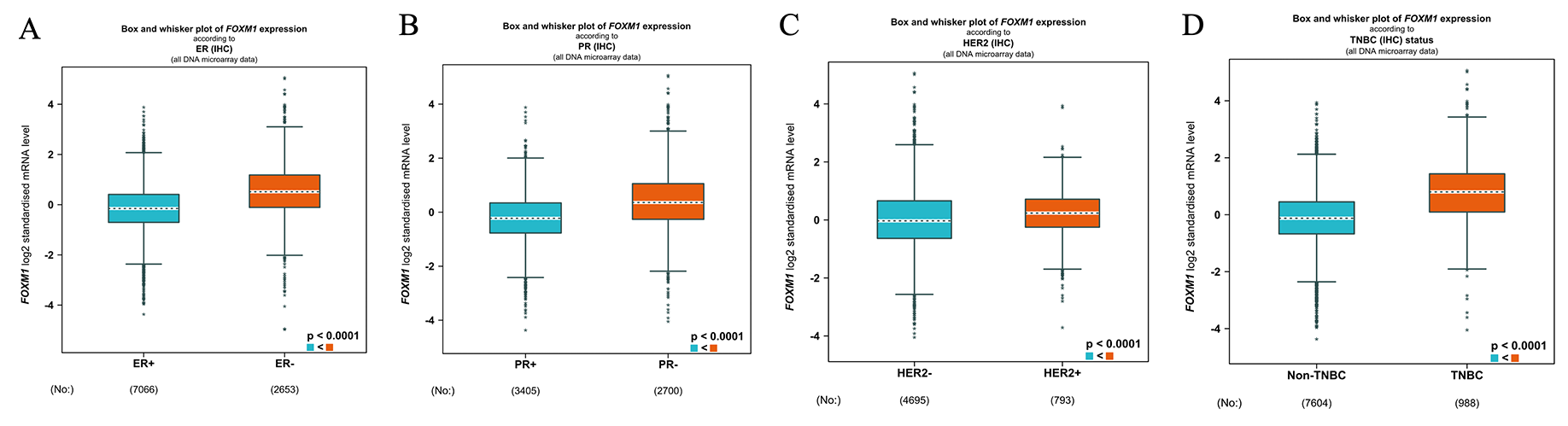

Supplement: Supplementary file 4 [file Image4.TIF]

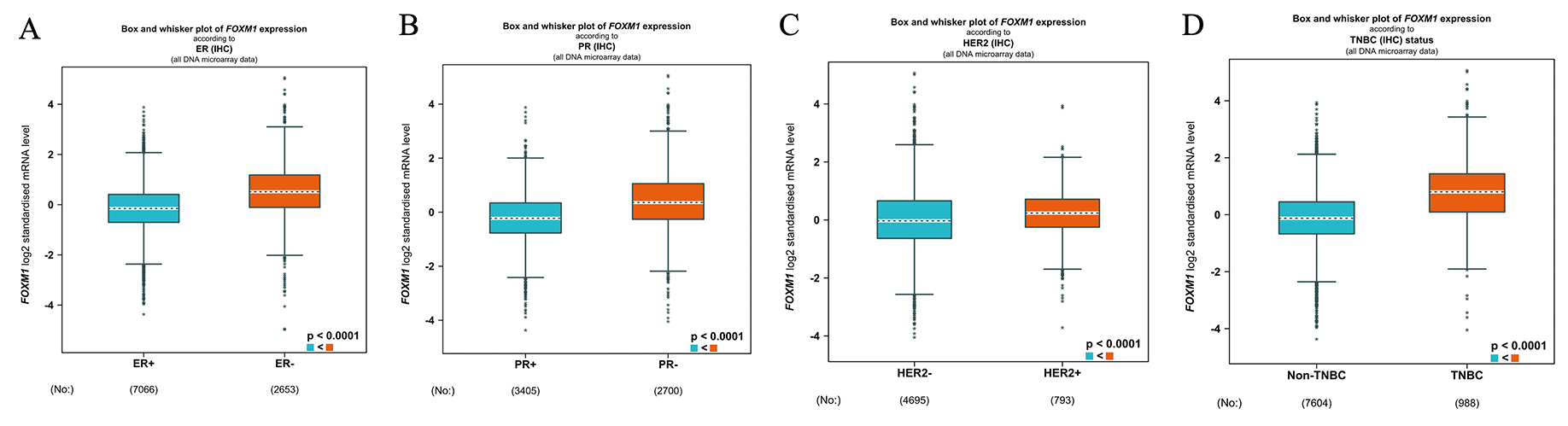

Supplement: Supplementary file 5 [file DataSheet1.zip › Supplementary file/Figure S4.tif]

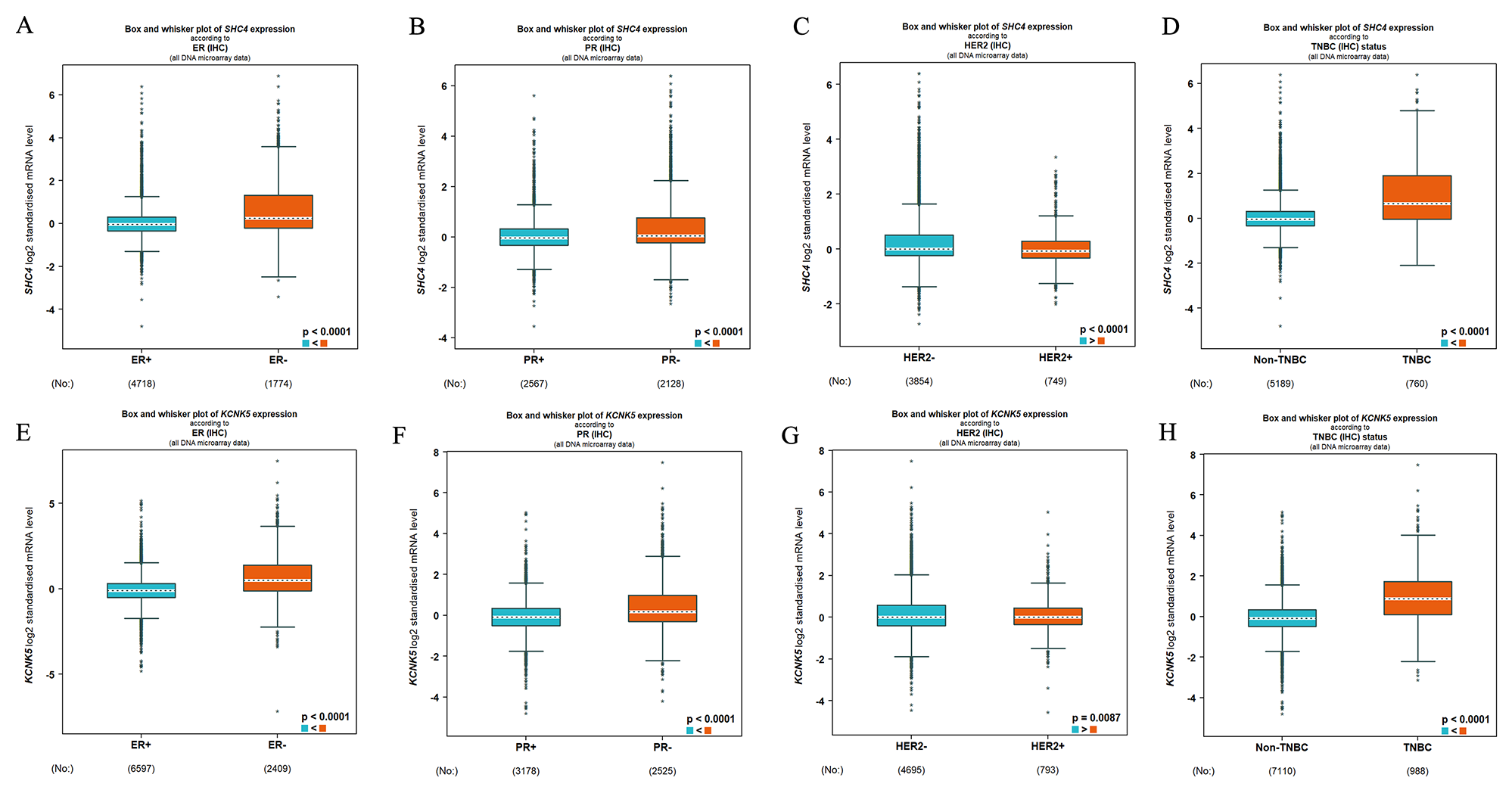

Supplement: Supplementary file 5 [file DataSheet1.zip › Supplementary file/Figure S2.tif]

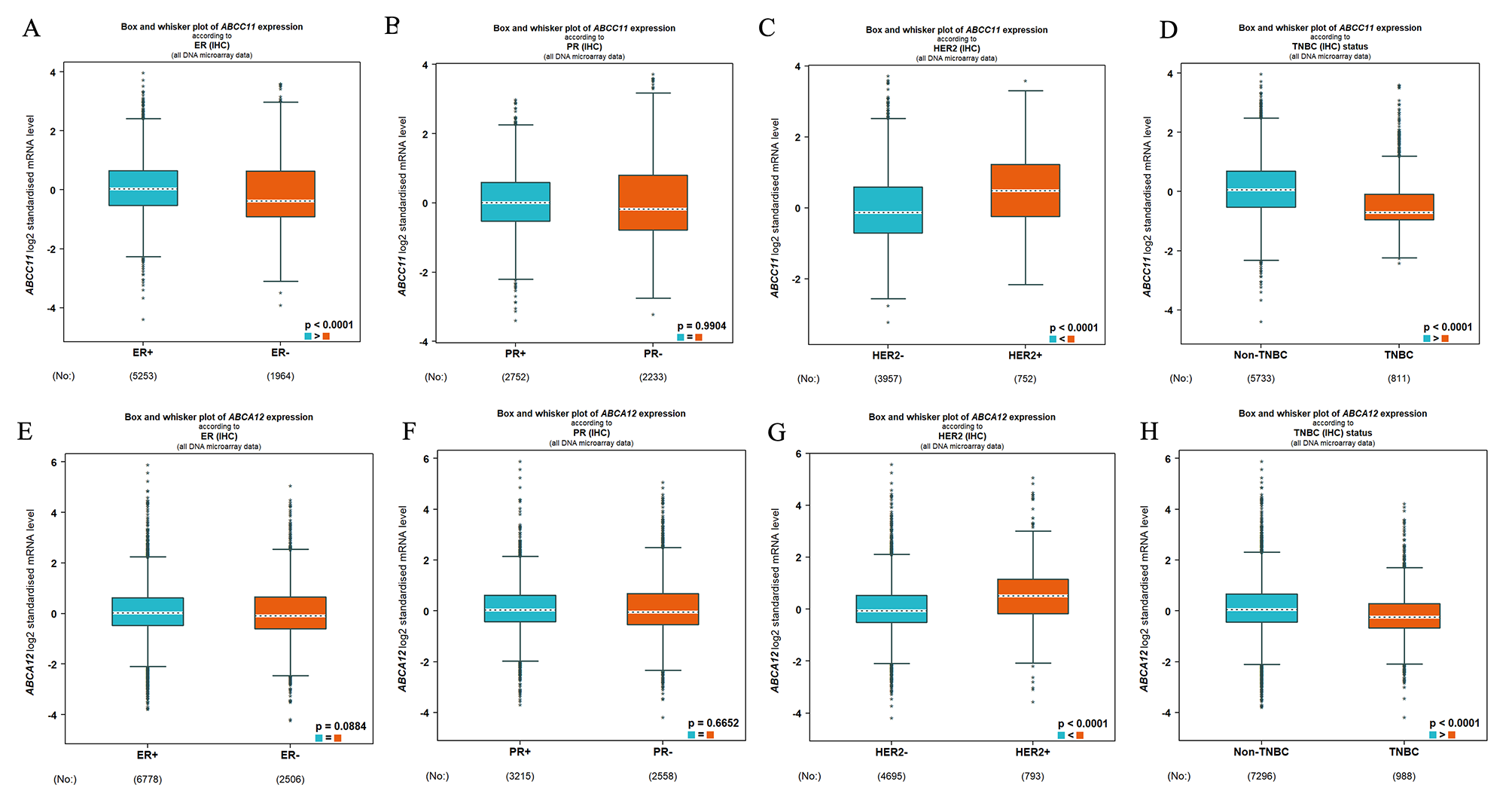

Supplement: Supplementary file 5 [file DataSheet1.zip › Supplementary file/Figure S3.tif]

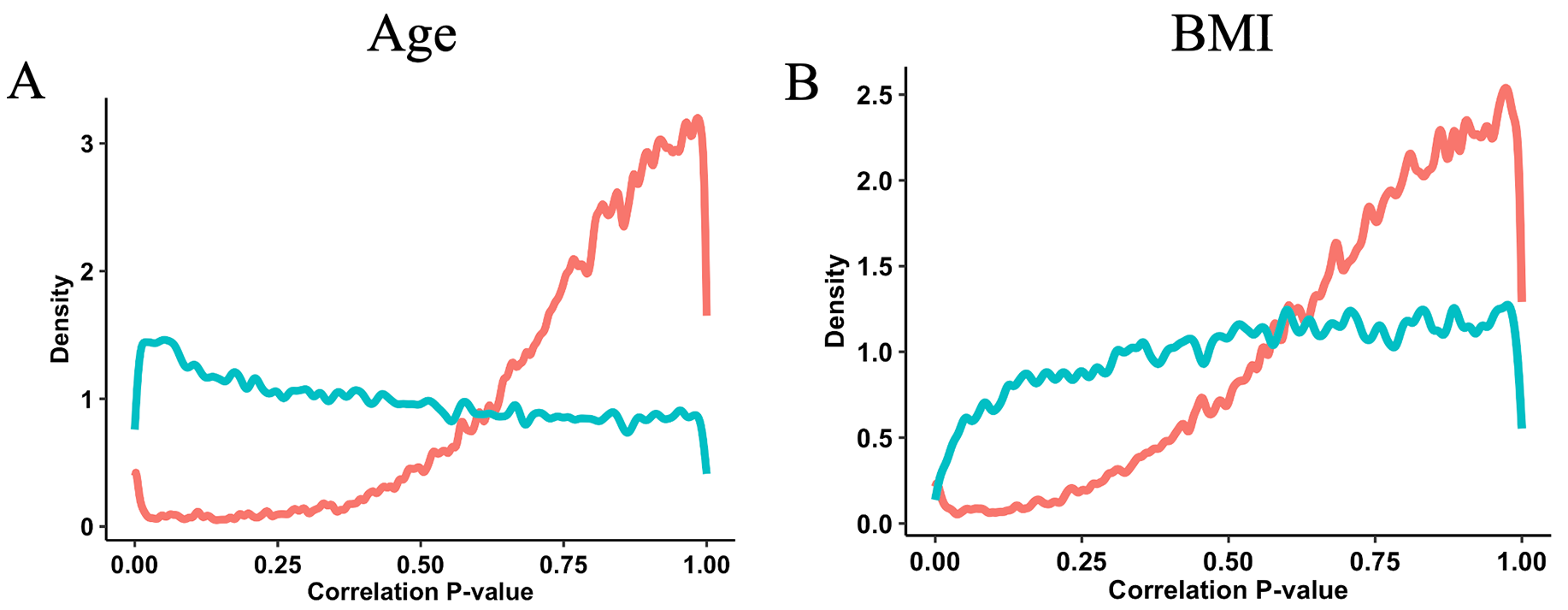

Supplement: Supplementary file 5 [file DataSheet1.zip › Supplementary file/Figure S1.tif]

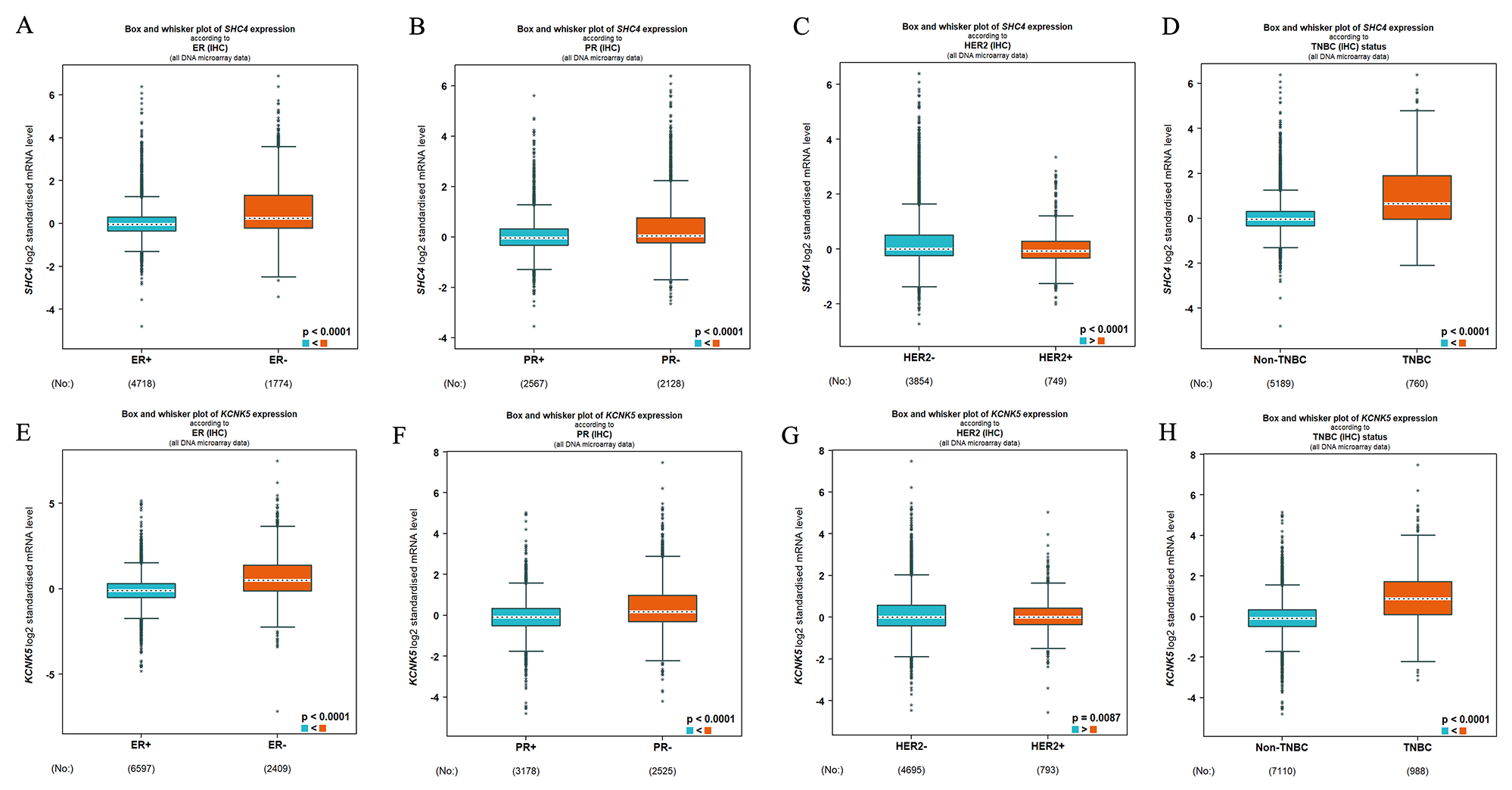

Supplement: Supplementary file 6 [file Image2.TIF]

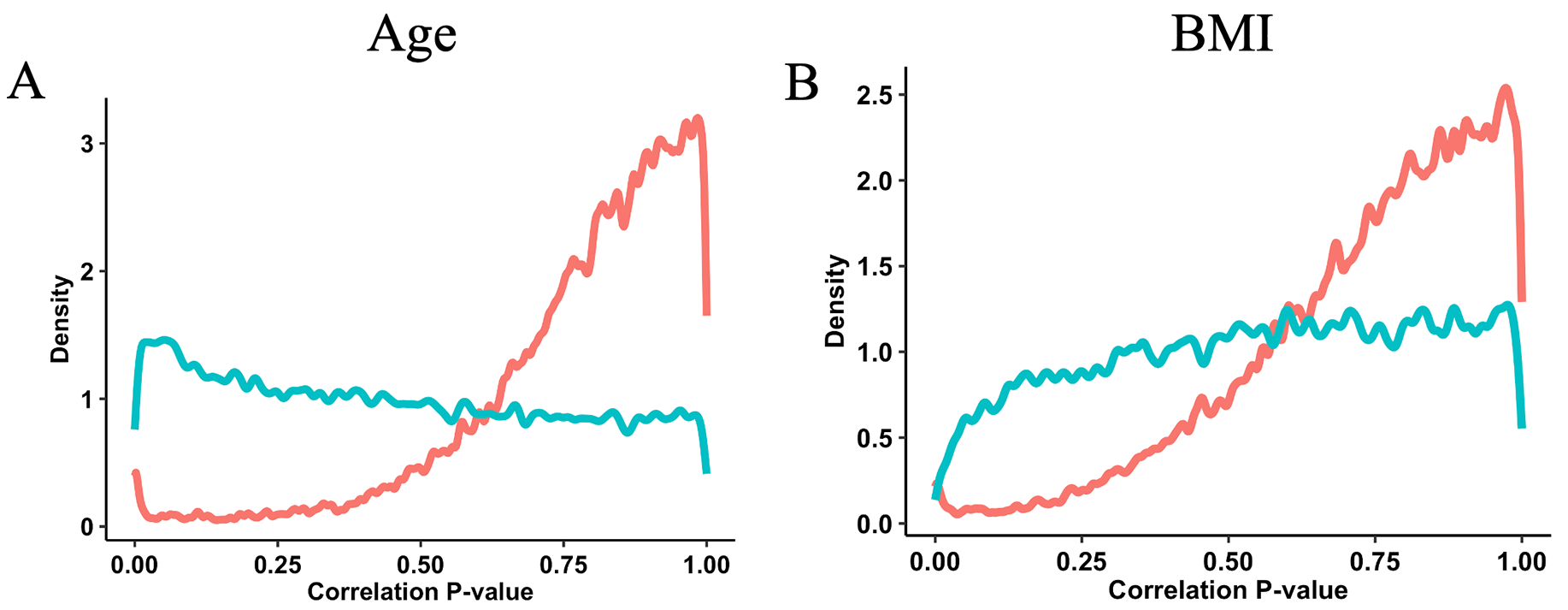

Supplement: Supplementary file 7 [file Image1.TIF]
